# Heterologous prime-boost: an important candidate immunization strategy against Tembusu virus

**DOI:** 10.1186/s12985-020-01334-w

**Published:** 2020-05-12

**Authors:** Yuting Pan, Renyong Jia, Juping Li, Mingshu Wang, Shun Chen, Mafeng Liu, Dekang Zhu, Xinxin Zhao, Ying Wu, Qiao Yang, Zhongqiong Yin, Bo Jing, Juan Huang, Shaqiu Zhang, Lin Zhang, Yunya Liu, Yanlin Yu, Bin Tian, Leichang Pan, Mujeeb Ur Rehman, Anchun Cheng

**Affiliations:** 1 Research Center of Avian Disease, College of Veterinary Medicine of Sichuan Agricultural University, Chengdu, 611130 People’s Republic of China; 2 Key Laboratory of Animal Disease and Human Health of Sichuan Province, Chengdu, 611130 People’s Republic of China; 3 Institute of Preventive Veterinary Medicine, Sichuan Agricultural University, Chengdu, 611130 People’s Republic of China

**Keywords:** Tembusu virus, E protein, Vaccine, Prime-boost strategy

## Abstract

**Background:**

Tembusu virus (TMUV), a newly emerging pathogenic flavivirus, spreads rapidly between ducks, causing massive economic losses in the Chinese duck industry. Vaccination is the most effective method to prevent TMUV. Therefore, it is urgent to look for an effective vaccine strategy against TMUV. Heterologous prime-boost regimens priming with vaccines and boosting with recombinant adenovirus vaccines have been proven to be successful strategies for protecting against viruses in experimental animal models.

**Methods:**

In this study, heterologous and homologous prime-boost strategies using an attenuated salmonella vaccine and a recombinant adenovirus vaccine expressing prM-E or the E gene of TMUV were evaluated to protect ducks against TMUV infection for the first time, including priming and boosting with the attenuated salmonella vaccine, priming and boosting with the recombinant adenovirus vaccine, and priming with the attenuated salmonella vaccine and boosting with the recombinant adenovirus vaccine. Humoral and cellular immune responses were detected and evaluated. We then challenged the ducks with TMUV at 12 days after boosting to assay for clinical symptoms, mortality, viral loads and histopathological lesions after these different strategies.

**Results:**

Compared with the homologous prime-boost strategies, the heterologous prime-boost regimen produced higher levels of neutralizing antibodies and IgG antibodies against TMUV. Additionally, it could induce higher levels of IFN-γ than homologous prime-boost strategies in the later stage. Interestingly, the heterologous prime-boost strategy induced higher levels of IL-4 in the early stage, but the IL-4 levels gradually decreased and were even lower than those induced by the homologous prime-boost strategy in the later stage. Moreover, the heterologous prime-boost strategy could efficiently protect ducks, with low viral titres, no clinical symptoms and histopathological lesions in this experiment after challenge with TMUV, while slight clinical symptoms and histopathological lesions were observed with the homologous prime-boost strategies.

**Conclusions:**

Our results indicated that the heterologous prime-boost strategy induced higher levels of humoral and cellular immune responses and better protection against TMUV infection in ducks than the homologous prime-boost strategies, suggesting that the heterologous prime-boost strategy is an important candidate for the design of a novel vaccine strategy against TMUV.

## Background

The genus *flavivirus* consists of single-stranded RNA viruses and includes more than 70 viruses, such as Tembusu virus (TMUV) [1], tick-borne encephalitis virus (TBEV) [2], dengue virus (DENV) [3], West Nile virus (WNV) [4], Japanese encephalitis virus (JEV) [5] and Zika virus (ZIKV) [6]. Flaviviruses can encode three structural proteins, capsid (C), premembrane/membrane (prM/M) and envelope (E), and seven nonstructural (NS) proteins, NS1, NS2A, NS2B, NS3, NS4A, NS4B and NS5. These proteins participate in viral invasion, replication and regulation of the host factors [7]. Flaviviruses can spread between animals and humans, causing zoonotic diseases [8].

TMUV is a newly emerging virus that is characterized by slow growth, decreased appetite, neurological dysfunction and a serious drop in egg production [9]. TMUV infects mainly ducks [10], chickens [11], geese [12], and pigeons [13]. Moreover, humans may also be threatened by TMUV infection [14]. Vaccination is the most effective method to prevent TMUV infection. Therefore, an effective vaccine immunization strategy against TMUV is urgently required. The E protein, which is the major antigenic determinant of the three structural proteins, contains many neutralizing epitopes and plays a critical role in host cell entry-attachment to cellular receptors and membrane fusion. Additionally, the E protein is the key region mediating viral virulence and is greatly immunogenic, which can induce immune protection effectively, suggesting that the E protein can be used as a vaccine candidate against TMUV [15]. The prM protein, which is regarded as the chaperone protein of E, can assist the E protein in proper folding and assembly and protect the structural stability of the E protein. Duck IL-2, a gene vaccine adjuvant, can strengthen the antigen-specific immune response of the vaccine and induce highly effective immunogenicity, which can provide the body with more comprehensive and efficient immune protection [16].

A homologous prime-boost regimen is used in traditional vaccines, but better preventive effects have been reported for infectious diseases by a heterologous prime-boost strategy, which consists of DNA vaccine priming followed by recombinant adenovirus boosting [17]. The heterologous prime-boost strategy can induce strong humoral and cellular immune responses [18, 19].

In the present study, vaccine strains using attenuated salmonella-presented TMUV prM-E gene [20] and recombinant adenovirus-packaged TMUV E gene with duck IL-2 as the vaccine adjuvant were successfully constructed. We wondered whether the heterologous prime-boost regimen (priming with attenuated salmonella vaccine and boosting with recombinant adenovirus vaccine) was more effective than the homologous prime-boost strategies (priming and boosting with the attenuated salmonella vaccine; priming and boosting with the recombinant adenovirus vaccine). Since development, attenuated *S. typhimurium* has been demonstrated as an effective safe carrier and is consequently the vector for delivering viral immunogenic genes [21, 22]. Additionally, the vaccine can be delivered orally, which is more convenient, economical and fast for large-scale clinical use. The replication-deficient adenovirus vector is highly immunogenic as a vaccine vector, which is safe for hosts [23–25]. On the other hand, the heterologous prime-boost strategy could induce better immune responses than homologous prime-boost strategy. Thus, this heterologous prime-boost strategy, which consists of priming with the attenuated salmonella vaccine and boosting with the recombinant adenovirus vaccine, is a promising choice for cost-effective mass vaccination. The results showed that the heterologous prime-boost strategy could induce higher levels of neutralizing antibodies and IgG antibodies and better protect ducks against TMUV infection than the homologous prime-boost regimens. Therefore, a heterologous prime-boost strategy should be carefully considered to induce the desired immune response against TMUV.

## Methods

### Virus and vaccines

The TMUV (CQW strain) used in this study was provided by the Research Center of Avian Disease of Sichuan Agricultural University. The virus was propagated in the allantoic cavities of 10-day-old specific pathogen-free (SPF) embryonated duck eggs. Vaccine strain using attenuated salmonella-presented TMUV prM-E (pVAX-SME) was kindly gifted by Huang [20]. (The attenuated *Salmonella typhimurium* aroA^−^ stain SL7207 (*S. typhimurium* 2337–65 derivative hisG46 DEL407 [aroA::Tn10 (Tcs)]) used in this attenuated salmonella vaccine was kindly provided by Professor Kai Schulze of the Helmholtz Center for Infection Research (Germany). The salmonella was attenuated by Tn10 transposon mutagenesis to generate deletion mutants in the aroA gene. Aro mutants are believed to be attenuated because they are unable to obtain essential aromatic metabolites in vivo [26, 27].) The recombinant adenovirus vaccine packaging the TMUV E gene with duck IL-2 as the vaccine adjuvant (rADV-E-IL-2) was saved in our laboratory. Construction of the recombinant adenovirus rADV-E-IL-2 was accomplished as follows. The fusion gene (E-IL-2) of 1733 bp that ligated the 1353 bp E gene of TMUV (GenBank: KM233707.1) and 350 bp duck IL-2 gene into the adenoviral shuttle vector by the RT-PCR method was cloned into the recombinant adenovirus shuttle plasmid to construct pshuttle-CMV-IRES2-EGFP-E-IL-2 (unpublished data). The positive control (a live TMUV vaccine named strain WF100) was purchased from Qilu Animal Health Company.

### Animal experiment

**Fig. 1:**
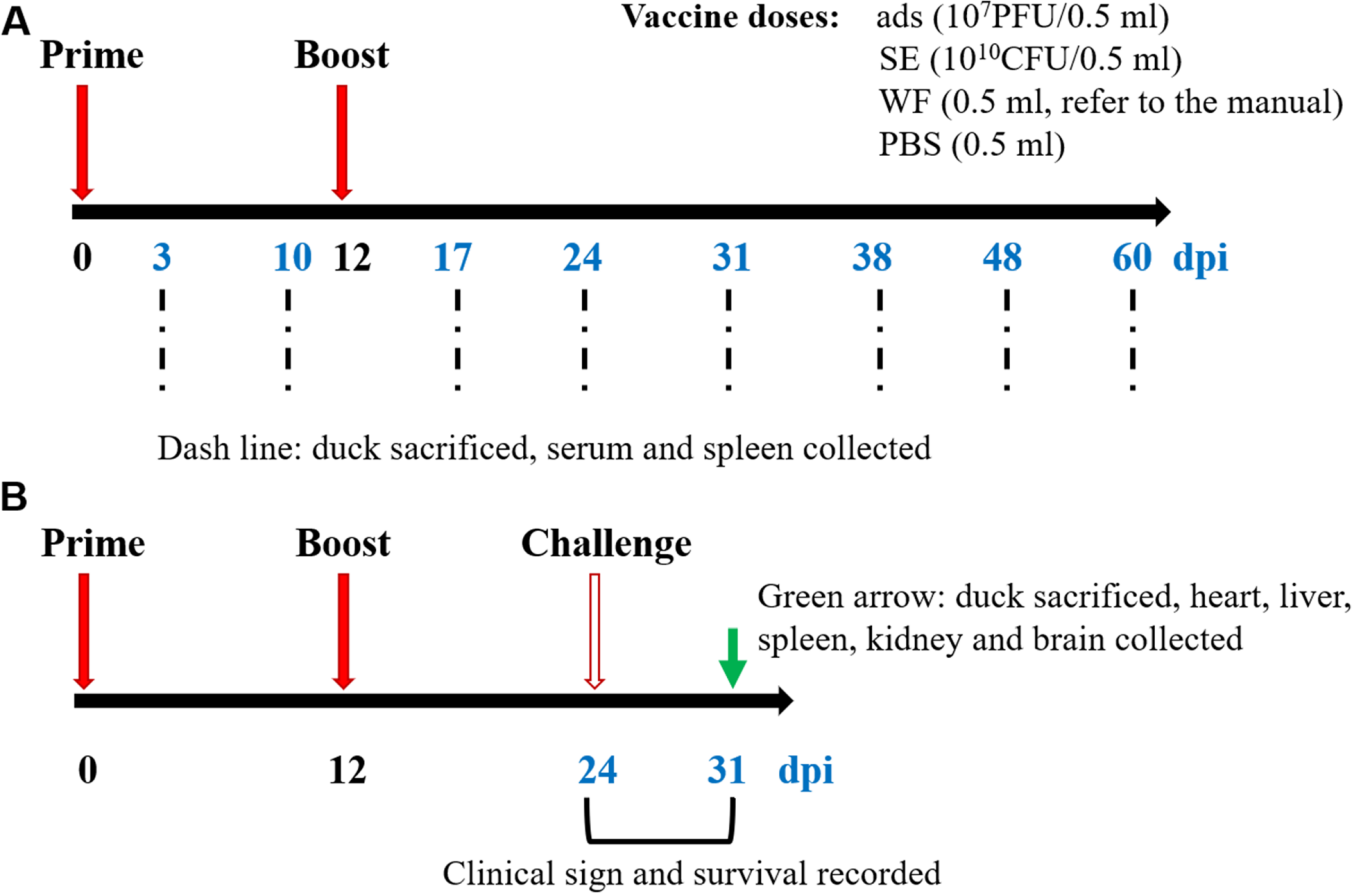
Duck experimental workflow. (**a**) Schedule of vaccination and sample collection. Ducks were vaccinated at 7 days old and 19 days old. Animals were sacrificed at 3, 10, 17, 24, 31, 38, 48 and 60 dpi (*n* = 3 of each time point); (**b**) Schedule of challenge experiment. The ducks (*n* = 10) of five groups were randomly selected at 12 days after the second immunization and challenged with 1 ml 10^5.1^ ELD_50_ TMUV to assay for the immune protection. The clinical signs and mortality were recorded for continuous 7 days after challenging

Ducklings (one day old) (*n* = 170) were purchased from a standard farm in Yaan and randomly divided into 5 groups, as shown in Table 1. All ducklings were obtained from flocks with no prior exposure to TMUV and were immunized twice, at 7 days old and 19 days old. At 3, 10, 17, 24, 31, 38, 48 and 60 days post prime injection (dpi), three ducks’ sera and spleens from each group were collected randomly and stored at − 80 °C (Fig. 1). Twelve days after boosting, ducks (*n* = 10) from each group were randomly selected and challenged with 1 ml of 10^5.1^-fold 50% of embryo lethal death (ELD_50_) TMUV per duck by intramuscular (i.m.) injection. Clinical symptoms and death were recorded until 7 days after challenge. Then, the heart, liver, spleen, kidney and brain of the ducks were collected and stored (Fig. 1).

**Table 1:** Experimental designs of the animal studies

Groups	Number of animals/ducks	Prime (7 days)			Boost (19 days)			Challenge (31 days)	
		Vaccine	Dosage	Route	Vaccine	Dosage	Route	Numbers of animals^j^	Challenge dosage
ads/ads^a^	34	ads^f^	10^7^PFU/0.5 ml	Intramuscular	ads	10^7^PFU/0.5 ml	Intramuscular	10	10^5.1^ELD_50_/1 ml
SE/SE^b^	34	SE^g^	10^10^CFU/0.5 ml	Oral injection	SE	10^10^CFU/0.5 m	Oral injection	10	10^5.1^ELD_50_/1 ml
SE/ads^c^	34	SE	10^10^CFU/0.5 ml	Oral injection	ads	10^7^PFU/0.5 ml	Intramuscular	10	10^5.1^ELD_50_/1 ml
WF/WF^d^	34	WF^h^	0.5 ml^i^	Intramuscular	WF	0.5 ml	Intramuscular	10	10^5.1^ELD_50_/1 ml
PBS/PBS^e^	34	PBS	0.5 ml	Intramuscular	PBS	0.5 ml	Intramuscular	10	10^5.1^ELD_50_/1 ml

### Neutralization assay

Serum samples from the immunized ducks were collected at 3, 10, 17, 24, 31, 38, 48 and 60 dpi, inactivated with heat at 56 °C for 1 h (*n* = 3 for each time point), serially 2-fold diluted to 2^− 8^ with phosphate buffer saline, mixed with an equal volume of 100 tissue culture infectious dose (TCID_50_) of TMUV and incubated in duck embryo fibroblast (DEF) cells with 5% CO_2_ at 37 °C for 1 h. Then, the mixture was removed, and Dulbecco’s modified Eagle media (DMEM) with 2% serum (Gibco, Gaithersburg, MD, USA) was added. The cells were cultured at 37 °C with 5% CO_2_ and observed to record the cytopathic effect (CPE) for at least 5 days. The titres of neutralizing antibodies against TMUV were determined according to the method of Reed and Muench by monitoring the CPE.

### Enzyme-linked immunosorbent assay

An enzyme-linked immunosorbent assay (ELISA) was used to detect the IgG antibody titres in the collected sera. TMUV E protein antigen (100 μl per well) as the capture molecule was coated in 96-well plates and incubated overnight at 4 °C. After washing the 96-well plates three times with phosphate-buffered saline with Tween (PBST), the plates were blocked with 1% BSA for 1 h at 37 °C. Horseradish peroxidase (HRP)-conjugated goat anti-duck IgG antibody (Solarbio, China) was used as the secondary antibody at a 1:2000 dilution for 1 h at 37 °C. After washing the plates three times with PBST, tetramethylbenzidine (TMB) (100 μl per well) was added to the plates in the dark for 10 min, and the reaction was stopped by 100 μl of 2 M H_2_SO_4_. Then, the optical density was measured in each sample at 450 nm. The result was calculated using the curve equation for sample concentration, and the critical value of IgG in serum was calculated by negative serum as previously described [28].

### Quantification of cytokines

Total RNA of the spleen collected from immunized ducks was extracted using TRIzol reagent (Invitrogen, Carlsbad, CA, USA) according to the manufacturer’s instructions. cDNA was synthesized from 1000 ng of RNA using a PrimeScript II 1st Strand cDNA Synthesis Kit with oligo dT primers (Takara Biotechnology, Dalian, China) according to the manufacturer’s instructions. Then, an equal volume of cDNA was subjected to quantitative real-time polymerase chain reaction (RT-PCR) using SYBR Green Real-Time RT-PCR Master Mix Plus (Promega) and primers. The primers for cytokine genes and β-actin used in this study are listed in Table 2. The quantitative RT-PCR procedure was performed as previously described [29]. The results were analysed by Bio-Rad CFX Manager software and GraphPad 7.0. by the 2^−∆∆Ct^ method and expressed as the mean ± standard deviation.

**Table 2:** List of primers and sequences in this study

Primer names	Polarity	Sequence (5′ - 3′)	Reference
IFN-γ(f)	Forward	CATACTGAGCCAGATTGTTACCC	Current study
IFN-γ(r)	Reverse	TCACAGCCTTGCGTTGGA	
IL-4(f)	Forward	TCTATCAGAGAAAGACAACAC	Current study
IL-4(r)	Reverse	GGTGACTATTTCTTTCAAGT	
β-actin(f)	Forward	CCGTGACATCAAGGAGAA	[29]
β-actin(r)	Reverse	GAAGGATGGCTGGAAGAG	
TMUV-C(f)	Forward	AGGTTTGTGCTGGCTCTAC	[30]
TMUV-C(r)	Reverse	TGTTTGGTCGCCTCATT	

### Detection of viral loads in challenged ducks by quantitative RT-PCR

Viral loads in tissues (heart, liver, spleen, kidney and brain) were detected by quantitative RT-PCR according to the method established previously [30]. Primers for the TMUV C protein are listed in Table 2. The results were analysed by Bio-Rad CFX Manager software and GraphPad 7.0. and expressed as the mean ± standard deviation.

### Pathological identification and histological examination

The tissues (heart, liver, spleen, kidney and brain) were fixed with 4% paraformaldehyde solution for at least 48 h, embedded in paraffin, and sliced into 4 μm sections (Leica RM2128, Wetzlar, Germany). The tissue sections were deparaffinized with xylene and rehydrated with gradient ethanol. Then, the sections were stained with haematoxylin and eosin (Solarbio, Beijing, China), counterstained with haematoxylin (Solarbio, Beijing, China), and dehydrated through a series of gradient ethanol solutions. We observed the samples with a microscope (Olympus BX43, Tokyo, Japan).

### Statistical analysis

Data and imaging were all performed by GraphPad 7.0. The relative mRNA expression was expressed as the mean ± standard deviation and analysed by the 2^−∆∆Ct^ method. The statistical significance was assessed by t-test. *p< 0.05 indicates significance.

## Results

### Specific antibody responses

**Fig. 2:**
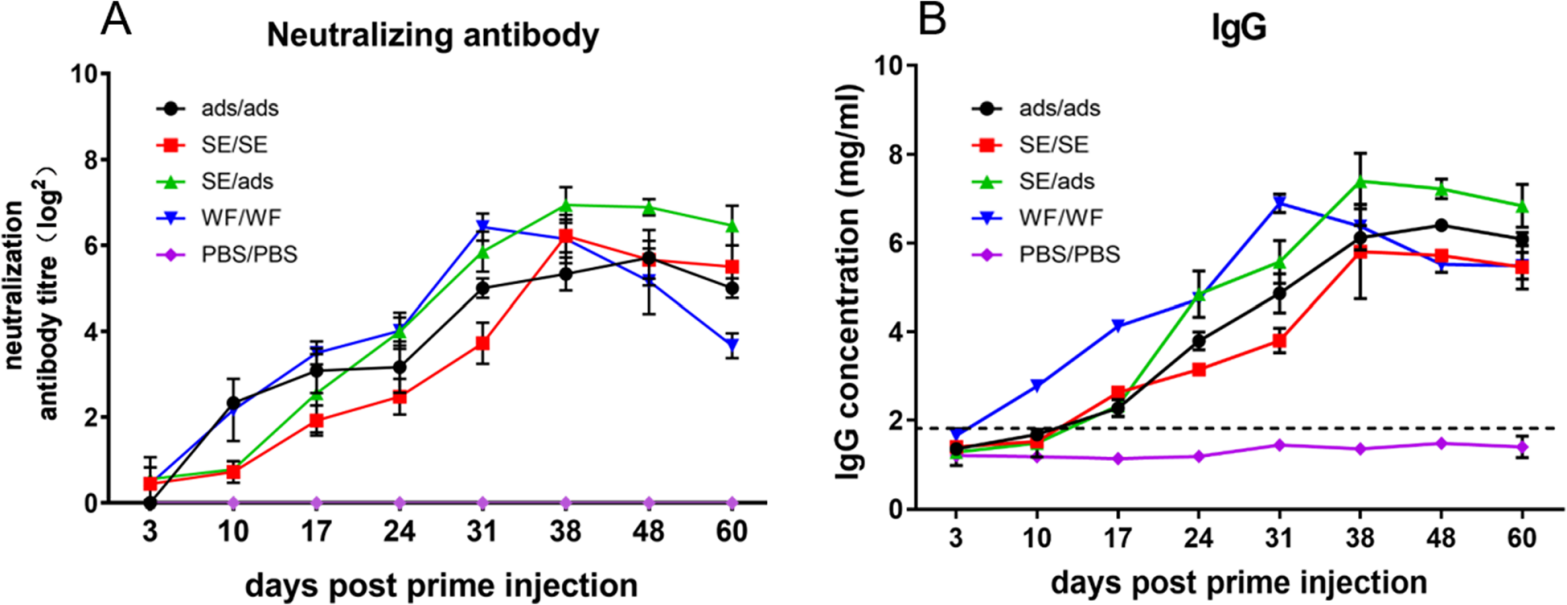
Production of antibodies. (**a**) Serum was collected from the ducks at 3, 10, 17, 24, 31, 38, 48 and 60 dpi. Gradient dilutions of serum and viral fluids were mixed and incubated with DEF cells for 1 h, then the mixture was removed, and DMEM with 2% serum was added. The DEF cells were cultured at 37 °C and observed for 5 days to record the lesions of cells. (**b**) The IgG antibodies in sera specific to the TMUV E protein were checked by ELISA. The serum samples collected from ducks were diluted and incubated with an E protein-coated plate. The specific anti-TMUV-E protein antibodies were measured by horseradish peroxidase-conjugated goat anti-duck IgG. The values of IgG are shown as the means ± standard deviations (*n* = 3 for each time point). The dashed line shows the detection limit for a positive response

To determine the protective immune responses induced by the heterologous or homologous prime-boost strategies, neutralizing antibodies were measured by a neutralization assay at 3, 10, 17, 24, 31, 38, 48 and 60 dpi. The neutralizing antibody levels of the ads/ads and SE/SE groups gradually increased and reached a peak at 38 dpi and 48 dpi with 5.7 log_2_-fold and 6.2 log_2_-fold increases, respectively (Fig. 2a). For the SE/ads group, the level of neutralizing antibody reached a peak at 38 dpi, with 6.9 log_2_-fold, and maintained a high level after the peak. The peak of the SE/ads group was 2.4 and 1.4 times higher than those of the ads/ads and SE/SE groups, respectively. The overall level of neutralizing antibodies in the SE/ads group was higher than those of the ads/ads and SE/SE groups. For the WF/WF group, the levels of neutralizing antibodies reached a peak at 31 dpi, with 6.4 log_2_-fold, but decreased quickly after the peak. There were no neutralizing antibody responses in the serum of ducks from the PBS/PBS group (Fig. 2a).

A high level of specific antibodies against the E protein in the SE/ads group was observed, which showed a 5.6-fold higher level at 38 dpi compared with that in the PBS/PBS group (Fig. 2b). The antibodies in the SE/ads group remained at high levels from 38 to 60 dpi and were higher than those in the ads/ads and SE/SE groups from 24 to 60 dpi. For the WF/WF group, the antibody titre reached a peak at 31 dpi, which was 4.93-fold higher than that in the PBS/PBS group, but decreased quickly after the peak and was even lower than that in the SE/ads group after 31dpi (Fig. 2b).

The results herein showed that the heterologous prime-boost strategy could efficiently induce the production of higher titres of antibodies in ducks than the homologous prime-boost strategies.

### Cytokines induced by the different immune strategies after immunization

**Fig. 3:**
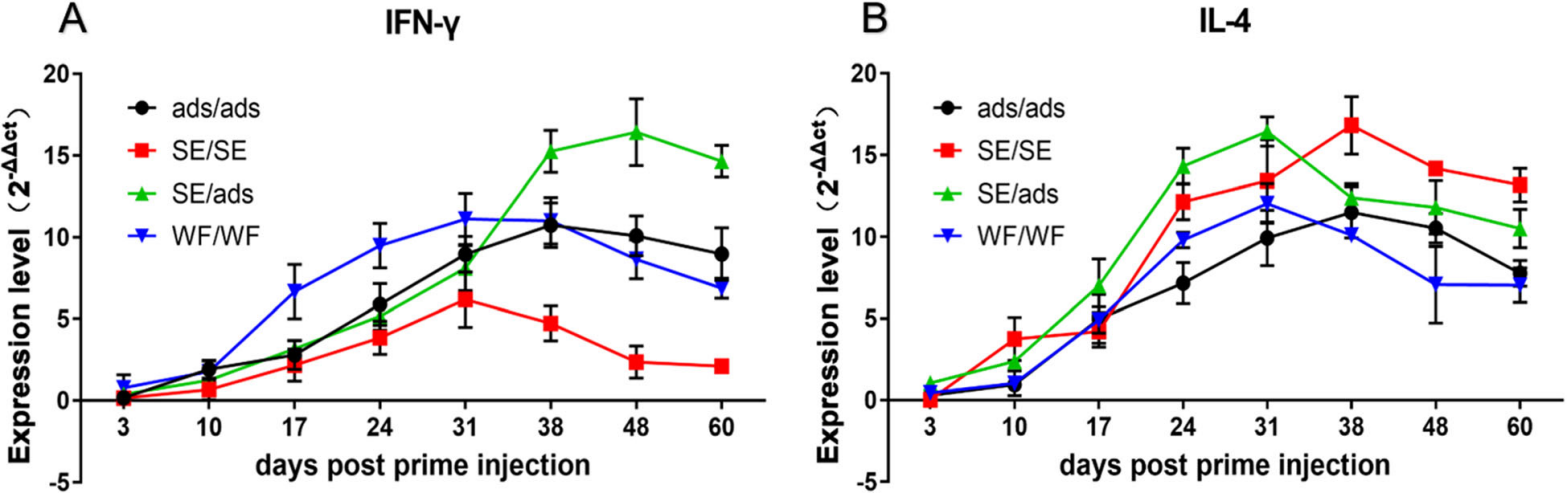
Comparative analysis the expression level of IFN-γ and IL-4 of the immunized ducks. The samples (spleen) were collected at 3, 10, 17, 24, 31, 38, 48 and 60 dpi to analyze the levels of cytokines by quantitative RT-PCR

To further characterize the induced immune response, quantitative RT-PCR was used to measure the cytokines (IFN-γ and IL-4) in the spleen. The expression level of IFN-γ in the ads/ads and SE/SE groups reached a peak that was 10.7-fold and 4.7-fold higher at 38 dpi and 31 dpi, respectively. For the SE/ads group, the expression level of IFN-γ increased quickly and exceeded that in the other three groups at 31 dpi. Then, it reached its peak at 48 dpi, which was 16.4-fold higher and maintained a high level of IFN-γ from 38 to 60 dpi. The expression level of IFN-γ in the WF/WF group, which reached a peak at 31 dpi with a 11.1-fold increase, was generally higher than that in the ads/ads and SE/SE groups, but lower than that in the SE/ads group after 31 dpi (Fig. 3a).

For IL-4, the expression levels of IL-4 in the ads/ads and SE/SE groups both reached peaks of 11.5-fold and 16.8-fold higher at 38 dpi, and the expression levels were lower than those in the SE/ads group from 17 to 31 dpi. Interestingly, the expression levels of IL-4 in the SE/ads group reached a peak at 31 dpi, which was 16.4-fold higher, but slowly decreased after the peak, and the levels were even lower than those in the SE/SE group after 31 dpi. For the WF/WF group, the expression level of IL-4 was generally lower than those in the SE/SE and SE/ads groups and lower than that in the ads/ads group after 31 dpi (Fig. 3b).

Collectively, these results demonstrated that the heterologous prime-boost strategy resulted in stronger stimulation of IFN-γ (in the later stage) and IL-4 (in the early stage) after immunization than the homologous prime-boost strategies.

### Protection of ducks against TMUV after challenge

**Fig. 4:**
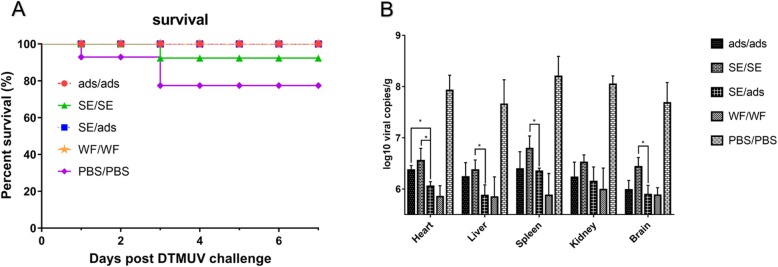
The protection afforded by vaccines against TMUV challenge. (**a**) Survival curves post challenge with TMUV. The immunized ducks (n = 10 per group) were challenged with 1 ml of 10^5.1^ ELD_50_ TMUV at 12 days after the second immunization. The survival was recorded for 7 consecutive days after challenge and graphed by GraphPad Prism v7.0. (**b**) Viral loads in tissues from ducks after challenge. Viral loads of tissues (heart, liver, spleen, kidney and brain) from each group were measured by quantitative RT-PCR. Data are expressed as the mean ± SD (n = 3). ‘*’ indicates a significant difference at *P* < 0.05

To verify the clinical protection of ducks against TMUV infection, the immunized ducks were challenged with 1 ml of 10^5.1^-fold ELD_50_ TMUV at 12 days after the boost vaccination (Table 1). All the ducks in the ads/ads, SE/ads and WF/WF groups survived. However, 10 and 30% of the ducks in the SE/SE and PBS/PBS groups died after challenge (Fig. 4a). Thus, the percent survival in the SE/SE and PBS/PBS groups was 90% (9/10) and 70% (7/10), respectively, while the percent survival in the ads/ads, SE/ads and WF/WF groups was 100% (10/10). To compare the protection against TMUV of these groups, the clinical signs were also recorded to evaluate the efficiency of these strategies. The ducks in the PBS/PBS group showed typical clinical signs after challenge, such as reluctance to move, head hung low, decreased appetite and neurological symptoms. However, slight clinical signs were also observed in the ads/ads and SE/SE groups, while no clinical signs were observed in the SE/ads and WF/WF groups. These results indicated that the heterologous prime-boost strategy provided better protection of the ducks against TMUV challenge than the homologous prime-boost strategies.

### Viral loads in the tissues of ducks after challenge

The titres of TMUV were measured by quantitative RT-PCR based on the C gene of TMUV at 7 days after challenge in the heart, liver, spleen, kidney and brain. TMUV could be detected in all tissues from the five groups after challenge. The viral titres of the spleen were higher than those of the other tissues (heart, liver, kidney and brain) (Fig. 4b). The titres of TMUV in the SE/ads group were generally lower than those of the ads/ads and SE/SE groups in the heart, liver, spleen, kidney and brain. In the heart, the titres of TMUV in the SE/ads group were significantly lower than those in the ads/ads and SE/SE groups (*P*<0.01). In the liver, spleen and brain, the viral titres in the SE/ads group were significantly lower than those in the SE/SE group (*P*<0.01), but there was no significant difference from those in the ads/ads group. In the kidney, the titres of TMUV in the ads/ads, SE/SE and SE/ads groups were not significantly different from each other (Fig. 4b). In summary, the heterologous and homologous prime-boost regimens could both inhibit viral replication after challenge in ducks, but the heterologous prime-boost regimen could prevent viral replication better than the homologous prime-boost strategies.

### Histopathological observation

**Fig. 5:**
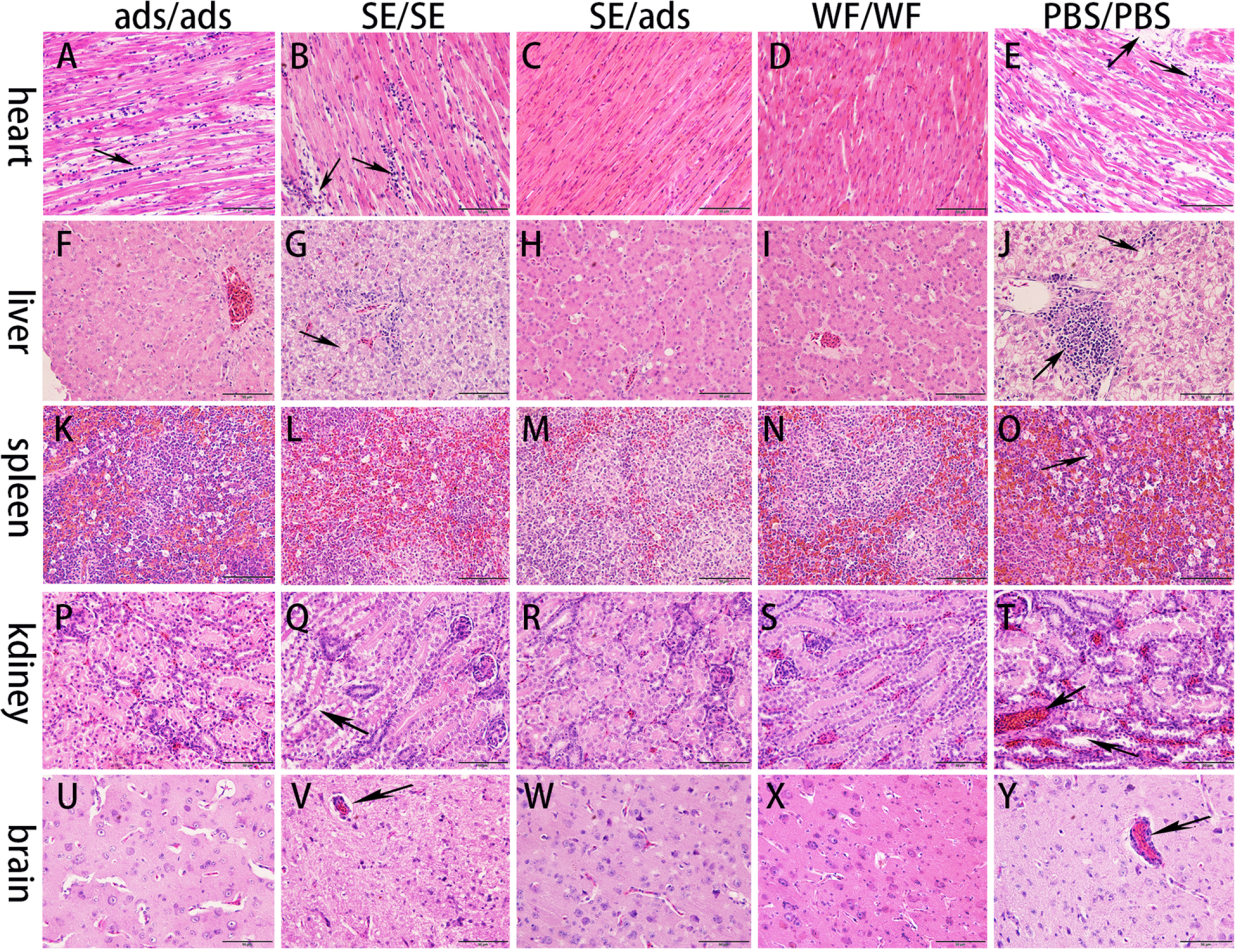
Histological lesions of the tissues after challenging with TMUV (400x). There were no histological lesions in the SE/ads and WF/WF groups, slight lesions in the ads/ads and SE/SE groups, and the most severe lesions were observed in the PBS/PBS group

The ducks were euthanized at 7 days after challenge, and their tissues (heart, liver, spleen, kidney and brain) were collected for histopathological observation. Histological observation of the different groups showed different histopathological changes. In the heart, slight myocardial fibre rupture and lymphocytic infiltration were found in the ads/ads and SE/SE groups (Fig. 5a and b), while no significant pathological damage was observed in the SE/ads and WF/WF groups (Fig. 5c and d). However, severe lesions in the PBS/PBS group were found with myocardial fibre rupture, oedema and lymphocytic infiltration (Fig. 5e).

In the liver, slight hepatocyte vacuolation was observed in the ads/ads group (Fig. 5f). Hepatocyte vacuolation and lymphocyte cell infiltration were observed in the SE/SE group (Fig. 5g), while the SE/ads and WF/WF groups had no obvious lesions (Fig. 5h and i). For the PBS/PBS group, the liver revealed severe hepatocyte vacuolation, hepatocyte necrosis and massive lymphocyte infiltration (Fig. 5j).

In the spleen, an obvious decrease in lymphocytes, an increase in reticulocytes and an unclear boundary between the red and white pulp were found in the ads/ads, SE/SE and PBS/PBS groups, while the other groups showed no significant lesions (Fig. 5k-o).

In the kidney, no obvious pathological changes were observed in the ads/ads, SE/ads and WF/WF groups (Fig. 5p, r and s). The basement membranes of renal epithelial cells in the SE/SE and PBS/PBS groups were obviously detached (Fig. 5q and t). Moreover, the kidney also experienced haemorrhage and necrosis in the epithelial cells of the PBS/PBS group (Fig. 5t).

In the brain, there were no obvious pathological changes in the ads/ads, SE/ads and WF/WF groups (Fig. 5u, w and x). The vascular sleeve phenomenon occurred in the brain tissues of the SE/SE group, while the PBS/PBS group showed both vascular sleeve and satellite phenomena (Fig. 5v and y).

In summary, there were slight lesions in the ads/ads and SE/SE groups and no histological lesions in the SE/ads and WF/WF groups, and the most severe lesions were observed in the PBS/PBS group.

## Discussion

Traditionally, the prime-boost strategy is performed using the same vaccine. However, new studies have suggested that the prime-boost strategy can be performed with different vaccines expressing the same antigen [31–34]. This type of heterologous prime-boost strategy has been used extensively in studies of vaccines against many pathogens, including human immunodeficiency virus (HIV), hepatitis C virus (HCV), pseudorabies virus and herpesvirus [35–37]. This fact may suggest that the heterologous prime-boost regimen can confer synergistically stronger responses to antigens and greater protection than immunization with the same vaccine alone [38, 39]. Based on our laboratory findings, vaccine strains using attenuated salmonella-presented TMUV prM-E and recombinant adenovirus-packaged TMUV E gene have been constructed. This paper describes the successful use of a heterologous prime-boost combination of attenuated salmonella presenting TMUV prM-E and recombinant adenovirus packaging the TMUV E gene for the first time to immunize ducks against TMUV infection.

Currently, the available vaccines against JEV, DENV and TBEV flavivirus infections can induce high levels of antibodies, which is deemed significant for protection after virus infection. The titres of antibodies are considered as a surrogate marker in vaccine evaluation [40–42]. In this study, the levels of IgG antibodies were detected after immunization with heterologous or homologous prime-boost strategies in ducks. The results showed that the heterologous prime-boost strategy could induce stronger antibody immunization than the homologous prime-boost regimens, especially after 31 dpi. This phenomenon also existed in neutralizing antibodies. The high levels of antibodies could greatly neutralize the free TMUV in the host and reduce the viral load in ducks.

One interesting result was that the cytokine levels induced by the heterologous prime-boost strategy were higher than those induced by the homologous prime-boost strategies in the early stage (17–31 dpi) for the IL-4 level (Th2-biased cytokine), and in the later stage (after 31dpi) for the IFN-γ level (Th1-biased cytokine), which may be the reason for the presence of the Th1-Th2 balance [43–45]. The expression levels of IFN-γ in the SE/ads group were inhibited by the high levels of IL-4 in the early stage, while the IL-4 levels in the SE/ads group were inhibited by the high levels of IFN-γ in the later stage. Thus, the IFN-γ levels in the SE/ads group increased slowly in the early stage, while the IL-4 levels decreased slowly in the later period. However, the levels of IL-4 in the SE/ads group were always higher than those in the ads/ads group at the eight time points. IFN-γ is an important immunoregulatory factor in the body that improves the body’s immune responses by mediating cellular immunity [46]. In this study, there was no significant difference in the expression level of IFN-γ between the heterologous or homologous prime-boost strategies in the early stage. However, in the later stage, the heterologous prime-boost strategy could induce higher levels of IFN-γ than the homologous prime-boost regimens, which could trigger a stronger cellular immune response in the later stage and better protect the ducks against TMUV.

Based on the protection against TMUV challenge, the ducks in the ads/ads group all survived after challenge. However, there were still slight clinical signs and lesions in the heart, liver and spleen when observed under a microscope. For the SE/SE group, a 90% survival rate in ducks was recorded after challenge with infectious TMUV, and slight lesions were observed in tissues, suggesting that the SE/SE group experienced only partial protection against TMUV. For the SE/ads group, this strategy could confer completely protection against TMUV infection with no death and no lesions in tissues. Although we detected high levels of antibodies in duck serum after immunization, the antibody levels in the testing groups were still rising after challenge. Since the level of neutralizing antibody had not reached a high level while challenging and the virus in ducks could not be completely eliminated, this result may be why the virus loads could still be detected at 7 days after challenge.

These results indicated that the heterologous prime-boost strategy was a better regimen than the homologous prime-boost regimens, as the SE/ads group exhibited substantial inhibition of TMUV replication in ducks and provided complete protection against TMUV infection with no death and no lesions in tissues, whereas the SE/SE and ads/ads groups exhibited only partial protection when vaccinated ducks were challenged.

The presented heterologous prime-boost regimen can be regarded as an alternative anti-TMUV vaccine strategy, as high titres of antibodies were induced in the blood, and all vaccinated animals survived with no clinical signs and no lesions in tissues after challenge. Discrimination between vaccinated and field-infected animals is possible due to the use of the prM and E proteins only in the vaccine. Finally, in our study, although the heterologous prime-boost strategy could cause a strong protective effect in animal experiments, safety evaluation is also necessary, as it is critical for every live vaccine to undergo a safety evaluation [47]. This research will also become the focus of our follow-up work, aiming at a more comprehensive evaluation of this heterologous prime-boost regimen.

In conclusion, this study described the first attempt to develop a novel immune strategy of a heterologous prime-boost regimen for TMUV based on the attenuated salmonella-presented TMUV prM-E and the recombinant adenovirus-packaged TMUV E gene. The results showed that the heterologous prime-boost strategy could produce great immunogenicity, and protect the ducks from the threat of TMUV infection. Therefore, the heterologous prime-boost regimen has great potential as a preventive strategy against TMUV infection.

## Conclusions

The heterologous prime-boost strategy could induce higher levels of antibodies and better protection against TMUV infection in ducks than the homologous prime-boost strategies, suggesting that the heterologous prime-boost strategy is an important candidate for the design of a novel vaccine strategy against TMUV.

## Data Availability

The data sets analyzed during the current study are available from the corresponding author on reasonable request.

## Abbreviations

TMUV: Tembusu virus
TBEV: Tick-borne encephalitis virus
DENV: Dengue virus
WNV: West Nile virus
JEV: Japanese encephalitis virus
ZIKV: Zika virus
C: Capsid
prM/M: Premembrane/membrane
E: Envelope
NS: Nonstructural
IL-2: Interleukin-2
SPF: Specific pathogen-free
ads: Recombinant adenovirus-packaged TMUV E gene with duck IL-2 as the vaccine adjuvant
SE: Attenuated salmonella vaccine-presented TMUV prM-E
WF: Positive vaccine named WF100 purchased from Qilu Animal Health Company
ads/ads: The homologous prime-boost immunization group that was primed with ads and boosted with ads
SE/SE: The homologous prime-boost immunization group that was primed with SE and boosted with SE
SE/ads: The heterologous prime-boost immunization group that was primed with SE and boosted with ads
WF/WF: The positive group that was primed with WF and boosted with WF
PBS/PBS: The negative group that was primed with PBS and boosted with PBS
dpi: Days post prime injection
ELD50: 50% of embryo lethal death
i.m.: Intramuscular injection
TCID50: Tissue culture infectious dose
DEF: Duck embryo fibroblast
DMEM: Dulbecco’s modified Eagle media
CPE: Cytopathic effect
ELISA: Enzyme-linked immunosorbent assay
IL-4: Interleukin-4
IFN-γ: Interferon-γ
RT-PCR: Real-time polymerase chain reaction
PBST: Phosphate buffer saline with Tween
HIV: Human immunodeficiency virus
HCV: Hepatitis C virus
